# Complex Epigenetic Regulation of Chemotherapy Resistance and Biology in Esophageal Squamous Cell Carcinoma via MicroRNAs

**DOI:** 10.3390/ijms19020499

**Published:** 2018-02-07

**Authors:** Kirsten Lindner, Ann-Kathrin Eichelmann, Christiane Matuszcak, Damian James Hussey, Jörg Haier, Richard Hummel

**Affiliations:** 1 Department of Endocrine Surgery, Schoen Kliniken, Dehnhaide 120, 22081 Hamburg, Germany; thurauki@googlemail.com; 2 Department of General and Visceral Surgery, University Hospital of Münster, Waldeyerstrasse 1, 48149 Münster, Germany; 3 University Cancer Centre Hamburg (UCCH), University Hospital of Hamburg-Eppendorf, Martinistr. 52 (O24), 20246 Hamburg, Germany; C.Matuszcak@uke.de; 4 Department of Surgery, Flinders Medical Centre, Flinders University Adelaide, Flinders Drive, Bedford Park, Adelaide, SA 5042, Australia; damian.hussey@flinders.edu.au; 5 The Nordakademie, Van-der-Smissen Str. 9, 22767 Hamburg, Germany; joerg.haier@nordakademie.de; 6 Department of Surgery, University Hospital of Schleswig-Holstein—Campus Lübeck, Ratzeburger Allee 160, 23538 Lübeck, Germany

**Keywords:** microRNA, esophageal squamous cell carcinoma, chemotherapy, resistance, metastasis, K410: KYSE-410, K270: KYSE-270, miR: miRNA

## Abstract

Background: Resistance towards chemotherapy is a major obstacle in the treatment of esophageal squamous cell carcinoma (ESCC). We investigated the role of specific microRNAs in chemotherapy resistance and tumor biology. Methods: We selected three microRNAs from characteristic microRNA signatures of resistant ESCC (hsa-miR-125a-5p, hsa-miR-130a-3p, hsa-miR-1226-3p), and hsa-miR-148a-3p. Effects on chemotherapy, adhesion, migration, apoptosis and cell cycle were assessed in six ESCC cell lines. Target analyses were performed using Western blotting and luciferase techniques. Results: MiR-130a-3p sensitized cells towards cisplatin in 100% of cell lines, miR-148a-3p in 83%, miR-125a-5p in 67%, miR-1226-3p in 50% (*p* ≤ 0.04). MiR-130a-3p sensitized 83% of cell lines towards 5-FU, miR-148a-3p/miR-125a-5p/miR-1226-3p only 33% (*p* ≤ 0.015). Several resistance-relevant pathways seem to be targeted on various levels. Bcl-2 was confirmed as a direct target of miR-130a-3p and miR-148a-3p, and p53 as a target of miR-125a-5p. All microRNAs decreased migration and adhesion, except miR-130a-3p, and increased apoptosis. Simultaneous manipulation of two microRNAs exhibited additive sensitizing effects towards cisplatin in 50% (miR-125a-5p/miR-148a-3p), and 75% (miR-148a-3p/miR-130a-3p) of cell lines (*p* ≤ 0.006). Conclusion: Our data present strong evidence that specific microRNA signatures are responsible for drug resistance and aggressiveness of ESCC. Final functional readout of these complex processes appears to be more important than single microRNA-target interactions.

## 1. Introduction

The overall prognosis of esophageal cancer (EC) is poor with five-year survival rates of 15–34% [1,2]. Neoadjuvant treatment plays a crucial role in management of advanced stage disease [1,2], but not all patients respond to this pre-treatment [3]. Furthermore, resistance to chemotherapy is often associated with more aggressive tumor biology. So far, no reliable biomarkers are available to evaluate patients’ individual response to neoadjuvant therapy or tumor biology.

MicroRNAs (miRNAs, miRs) are small non-coding single-stranded, endogenous RNA molecules that might be able to identify and maybe even modulate resistance to treatment. Development of resistance towards chemotherapeutics is complex and involves multiple aspects such as transmembrane transport of drugs [4], intracellular drug metabolism [5] or cellular proliferation and apoptosis [6,7,8,9,10,11]. MiRNAs regulate global gene expression post-transcriptionally [6] and control many fundamental cellular processes, including cell differentiation, cell proliferation, apoptosis, and metabolism [12,13,14,15]. Most importantly, miRNAs have clearly been linked to cancer [16] and to sensitivity to chemotherapy [15,17,18].

In our previous work, we were able to identify characteristic miRNA signatures that distinguish cisplatin or 5-FU resistant esophageal squamous cell carcinoma cells (ESCC) from sensitive control cells [19]. The aim of the current study was to investigate if these specific miRNA signatures of resistant cell lines are in fact responsible for their chemotherapy resistant phenotype. Moreover, as increasing resistance to chemotherapy clinically often correlates with more aggressive tumor behavior, we further questioned if these miRNA signatures impact biological behavior of cancer cells such as metastatic potential and ability to escape apoptosis. Finally, we aimed to investigate relevant intracellular pathways that might be regulated by these miRNAs and that might mediate their effects on chemotherapy resistance.

## 2. Results

### 2.1. Specific miRNA Signatures of Resistant ESCC Affect Chemotherapy Resistance

All selected miRNAs significantly affected chemotherapy resistance in ESCC cells.

Downregulation of miR-130a-3p increased sensitivity towards cisplatin in all six cell lines (sensitivity: +7% to +22%; *p* ≤ 0.002). Upregulation of miR-148a-3p induced better response to cisplatin (+13% to +28%; *p* ≤ 0.04) in five cell lines. Increased expression of miR-125a-5p sensitized four out of six cell lines towards cisplatin (+5% to +22%; *p* ≤ 0.002), and upregulation of miR-1226-3p increased sensitivity towards cisplatin (+8% to +18%; *p* ≤ 0.031) in three cell lines.

**Figure 1:**
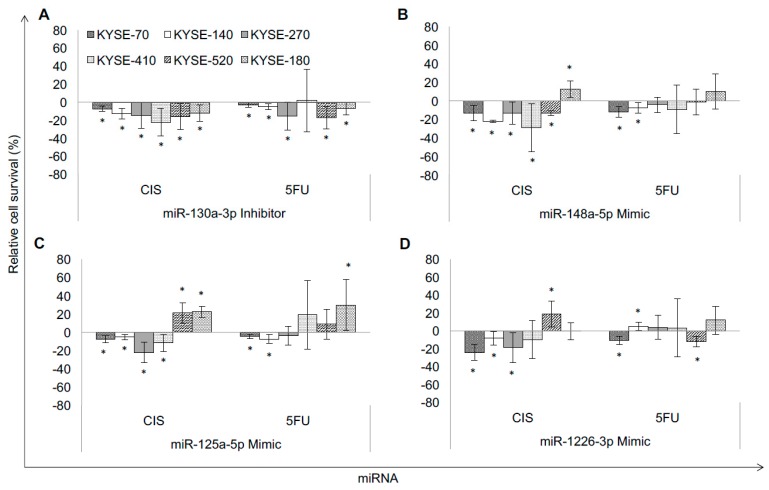
Specific miRNA signatures of resistant esophageal squamous cell carcinoma (ESCC) cell lines are responsible for chemotherapy resistant phenotypes. Effect of miRNA modulation on cytotoxicity after chemotherapy treatment: (**A**) miR-130a-3p Inhibitor; (**B**) miR-148a-5p Mimic; (**C**) miR-125a-5p Mimic; (**D**) miR-1226-3p Mimic. Relative cell survival compared to controls given in %, controls set to “zero”. K70: KYSE-70, K140: KYSE-140, K270: KYSE-270, K410: KYSE-410, K520: KYSE-520, K180: KYSE-180, CIS: cisplatin, 5-FU: 5-fluorouracil; * significance (*p* ≤ 0.05).

With regards to 5-FU, downregulation of miR-130a-3p increased sensitivity towards 5-FU in five out of six cell lines (sensitivity: +3% to +17%; *p* ≤ 0.002, Figure 1A). Upregulation of miR-148a-3p led to better response to 5-FU (+7% to +12%; *p* ≤ 0.002, Figure 1B) in two cell lines. Increased expression of miR-125a-5p sensitized two cell lines towards 5-FU (+5% to +7%; *p* ≤ 0.015, Figure 1C), and upregulation of miR-1226-3p increased sensitivity towards 5-FU (+11% to +12%; *p* ≤ 0.002, Figure 1D) in two out of six cell lines.

### 2.2. Co-Transfection: Synergistic Effects on Resistance to Cisplatin

Next, we evaluated a potential additive/opposing effect of different miRNAs on chemotherapy resistance focusing on three miRNAs that impacted on resistance towards cisplatin in the majority of cell lines: miR-130a-3p, miR-148a-3p and miR-125a-5p. We either upregulated expression of miR-125a-5p and miR-148a-3p simultaneously, or we downregulated miR-130a-3p expression with a simultaneous upregulation of miR-148a-3p.

**Figure 2:**
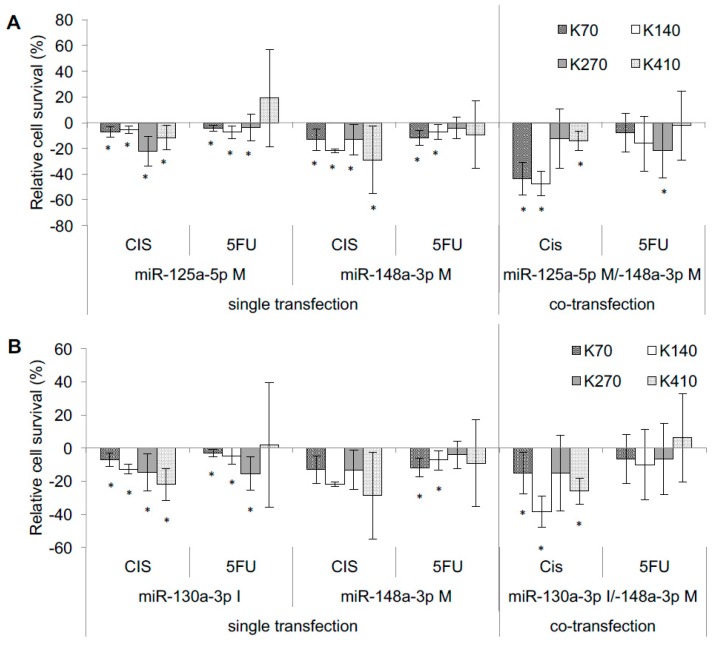
Co-Transfection: additive effects of resistance-relevant miRNAs. Effect of co-transfection of miRNAs on cytotoxicity after chemotherapy treatment for (**A**) miR-125a-5p Mimic/miR-148a-3p Mimic and (**B**) miR-130a-3p Inhibitor/miR-148a-3p Mimic, compared to single transfection controls with each miRNA. Relative cell survival compared to controls given in %, controls set to “zero”. K70: KYSE-70, K140: KYSE-140, K270: KYSE-270, K410: KYSE-410, CIS: cisplatin, 5-FU: 5-fluorouracil, M: mimic, I: inhibitor; * significance (*p* ≤ 0.05).

Co-transfection with miR-125a-5p/miR-148a-3p mimics resulted in significantly increased sensitivity towards cisplatin (+14% to +47%; *p* ≤ 0.006) in three out of four cell lines compared to scrambled controls, and we found an additive effect of co-transfection in two out of four experiments compared to transfections with either miRNA alone (Figure 2A).

Co-transfection of miR-148a-3p/miR-130a-3p resulted in significantly increased sensitivity towards cisplatin in all cell lines (+15% to +39%; *p* ≤ 0.005) compared to scrambled controls, and led in 75% of our experiments to an additive effect of co-transfection when compared to transfections with either miRNA alone (Figure 2B).

We did not observe a coherent effect of co-transfection on response to 5-FU treatment. While single miRNA transfections resulted in significant increases in sensitivity towards 5-FU in about 63% of all experiments, only miR-125a-5p/miR-148a-3p co-transfection for KYSE-270 led to significantly better response to 5-FU compared to single transfected controls and increased sensitivity by about 16%.

### 2.3. Specific miRNA Signatures of Resistant ESCC Impact on Metastatic Behaviour of ESCC 

**Figure 3:**
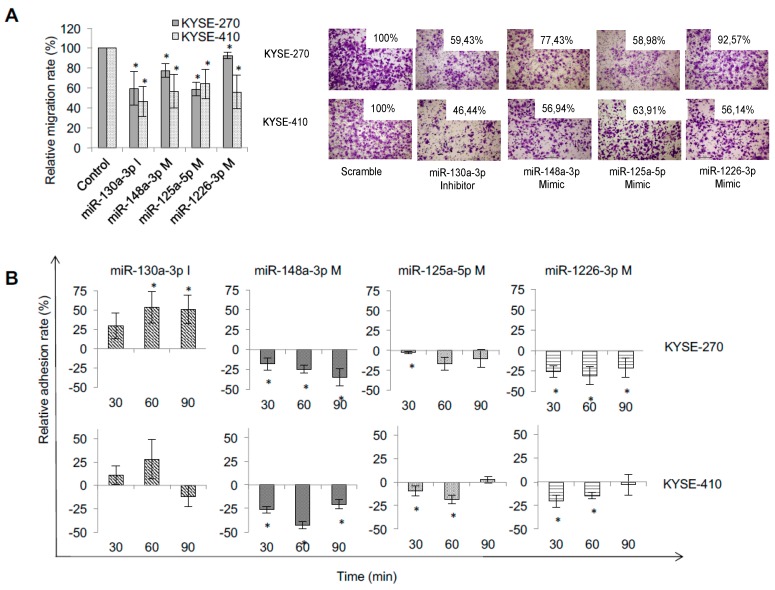
Specific miRNA signatures of resistant cell lines impact on metastatic behavior of ESCC. (**A**) Migration assay of transfected cells (200× magnification). K270: KYSE-270, K410: KYSE-410, M: Mimic, Inhib. +I: inhibitor; * significant difference (*p* ≤ 0.05); (**B**) effect of adhesion assay after transfection. M: mimic, I: inhibitor; * significance (*p* ≤ 0.05).

All four miRNAs significantly reduced cellular migration (*p* ≤ 0.01, Figure 3A), and three out of four miRNAs (namely miR-125a-5p, miR-148a-3p and miR-1226-3p; *p* ≤ 0.05) led to time-dependent delay in adhesion of tumor cells (Figure 3B).

### 2.4. Specific miRNA Signatures of Resistant ESCC Impact on Apoptosis and Cell Cycle

**Figure 4:**
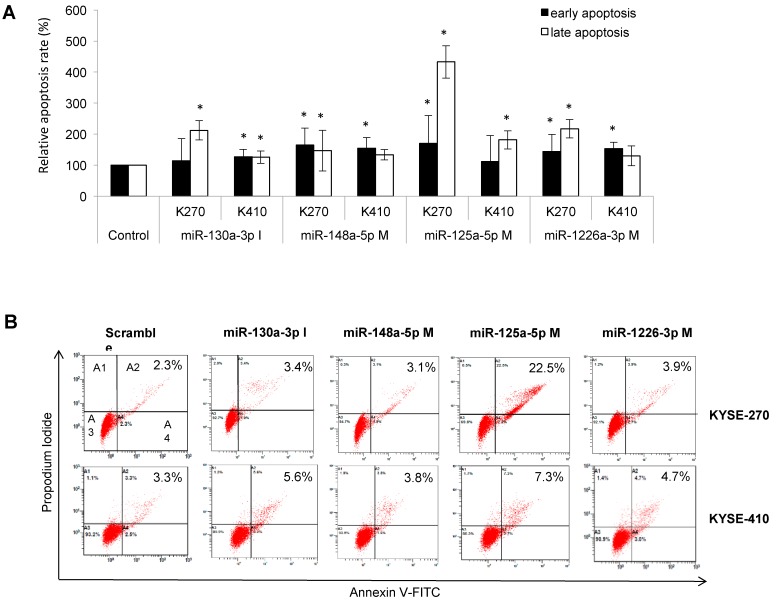
Specific miRNA signatures of resistant cell lines impact on apoptosis in ESCC. (**A**) Relative apoptosis rate of transfected cells vs. negative controls; (**B**) representative dot plot of the Annexin V-FITC and PI assay on ESCC cells treated with 20 ppmol of different miRNAs after 48 h of transfection. (A1: Necrotic cells/false positive cells (Annexin−/7AAD+); A2: Late apoptotic cells (Annexin+/7AAD+); A3: Viable cells (Annexin−/7AAD−); A4: Early apoptotic cells (Annexin+/7AAD−). K270: KYSE-270, K410: KYSE-410, M: mimic, I: inhibitor; A2: late apoptotic rate, A4: early apoptotic rate; * significance (*p* ≤ 0.05).

Altered expression of all four miRNAs significantly increased (especially late-) apoptosis rates with a maximum increase in apoptosis of up to 332% after miR-125a-5p upregulation (Figure 4).

**Figure 5:**
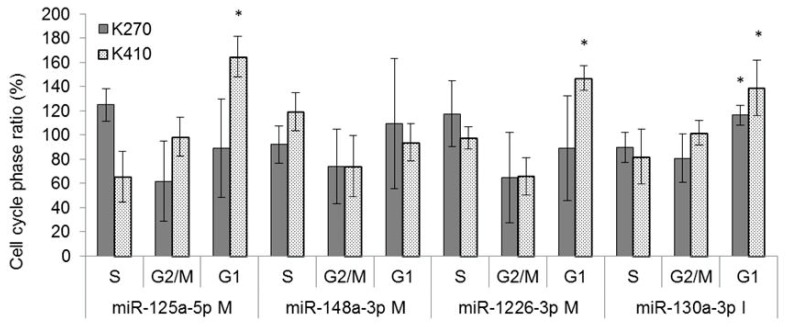
Specific miRNA signatures of resistant cell lines impact on cell cycle in ESCC. Cell cycle analysis of ESCC cells after transfection with respective miRNAs in KYSE-270 and KYSE-410. S: synthesis phase of DNA replication, G2: gap 2 between DNA synthesis and mitosis, M: mitosis phase, G1: gap 1 as beginning of interphase, K270: KYSE-270, K410: KYSE-410; * significance (*p* ≤ 0.05).

Furthermore, inhibition of miR-130a-3p resulted in G1 arrest, and upregulation of miR-125a-5p and miR-1226a-3p led to G1 arrest in KYSE-410, but not in KYSE-270 cells (Figure 5).

### 2.5. Specific miRNA Signatures of Resistant ESCC Target Various Resistance-Relevant Pathways 

To investigate downstream effects of the selected miRNAs, we analyzed a number of potential targets.

**Figure 6:**
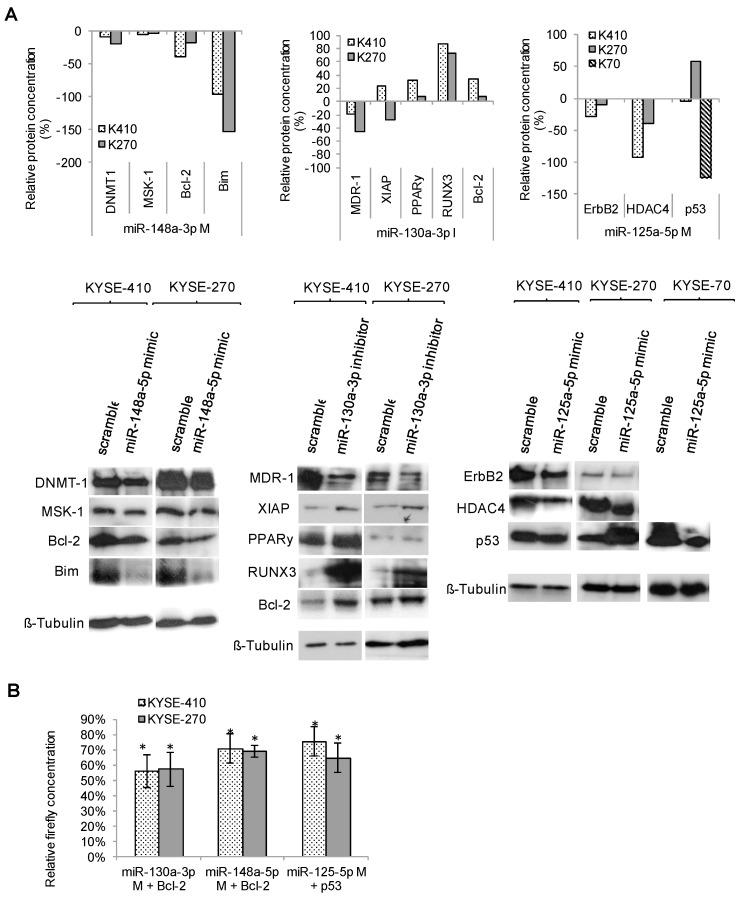
Specific miRNA signatures of resistant cell lines target various resistance-relevant pathways: Western blotting and luciferase assays. (**A**) Protein expression of potential targets of respective miRNAs, measured with Western blot in KYSE-410, KYSE-270 cells (and in case of p53 analysis in KYSE-70 cells); (**B**) luciferase assay after miRNAs transfection. Relative firefly concentration of target protein of interest was measured with the Dual Glo Luciferase Kit 24 h after transfection with miRNA precursor molecules. DNMT-1: DNA (cytosine-5)-methyltransferase 1; MSK-1: mitogen and stress activated protein kinase 1; Bcl-2: B-cell lymphoma 2; MDR1: multidrug resistance protein 1; XIAP: X-linked inhibitor of apoptosis protein; RUNX3: Runt-related transcription factor 3; PPARy: Peroxisome proliferator-activated receptor gamma; HDAC4: Histone deacetylase 4; ErbB2: Receptor tyrosine-protein kinase; K270: KYSE-270, K410: KYSE-410, K70: KYSE-70; M: mimic, I: inhibitor, Scr: Scramble; * significance (*p* ≤ 0.05).

Western blot analyses showed that upregulation of miR-125a-5p resulted in lower protein levels of ErbB2/Her2, HDAC4 and p53. Similarly, PPARγ, Bcl-2, XIAP and RUNX3 showed decreased protein levels after upregulation of miR-130a-3p. Finally, increased miR-148a-3p levels resulted in decreased protein levels of DNMT1, MSK-1, Bcl-2 and Bim (Figure 6A). 

Luciferase assays then proved Bcl-2 as a direct target of miR-130a-3p and miR-148a-3p, and p53 as a direct target of 125a-5p (Figure 6B).

**Figure 7:**
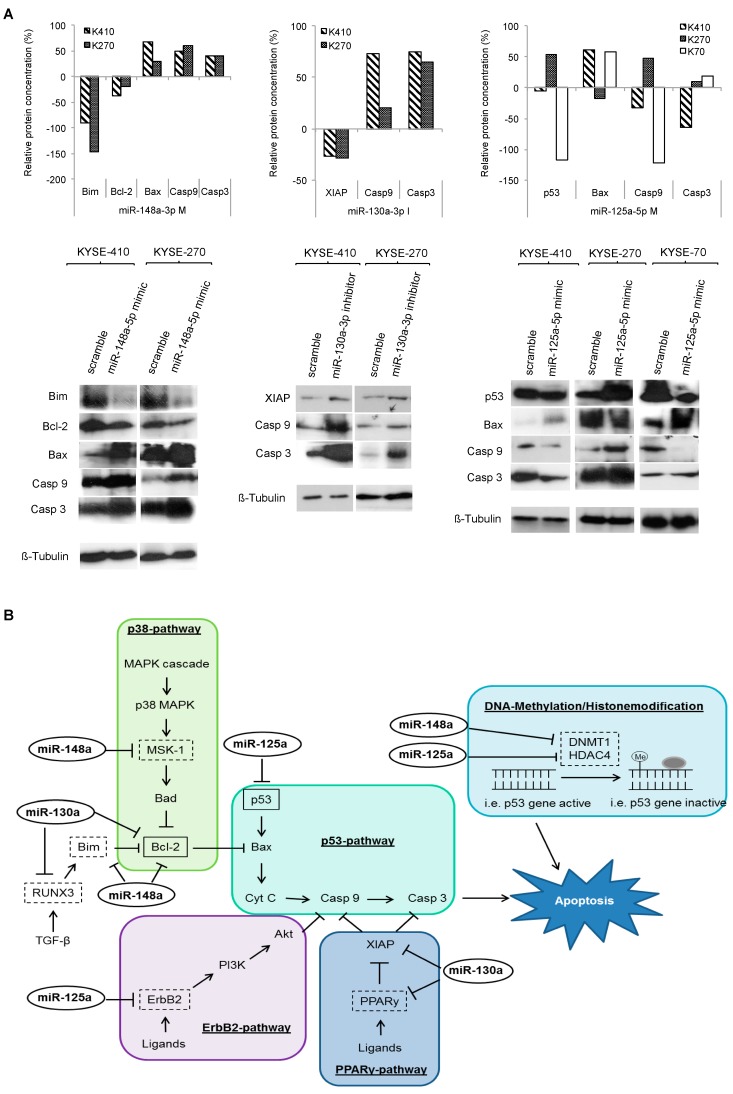
Specific miRNA signatures of resistant cell lines target various resistance-relevant pathways: pathway analyses. (**A**) Effect of miRNA transfection on the p53-dependant apoptosis pathway in ESCC cells. Relative protein expression levels of pathway compounds compared to controls were measured via Western blot analysis; (**B**) overview about the complex process of miRNA-mediated regulation of several resistance-relevant pathways at various key spots. Confirmed direct targets are marked in boxes with solid lines, potential targets are marked in boxes with dotted lines (adapted from Krammer et al. [20]); Bcl-2: B-cell lymphoma 2; Bax: Bcl-2-associated X protein; Casp: caspase; XIAP: X-linked inhibitor of apoptosis protein; K270: KYSE-270, K410: KYSE-410, K70: KYSE-70; M: mimic, I: inhibitor, Scr: Scramble.

As many of these targets regulate terminal apoptotic signaling, we analyzed the expression of further downstream steps within the apoptotic pathway including Bax, caspase 3 and 9 after transfection with the respective miRNAs. We found that all three miRNAs modulate expression of their targets as well as of the entire apoptotic pathway down to the caspases (Figure 7A).

## 3. Discussion

Resistance towards chemotherapy and aggressive tumor biology severely impacts outcomes in EC patients. Identification of treatment responders or prognostic unfavorable disease seems of utmost importance to allocate patients to optimal individualized treatment. The identification of characteristic miRNA expression signatures in resistant ESCC in our previous studies suggested that miRNAs might play a key role in epigenetic regulation of chemotherapy resistance and tumor biology in EC [21,22,23].

Our data now provide strong evidence that characteristic miRNA signatures of resistant ESCC are in fact responsible for the chemotherapy resistant phenotype of this tumor. By analyzing four miRNAs in six ESCC cell lines, we could show that miR-125a-5p, miR-130a-3p, miR-148a-3p and miR-1226-3p significantly increased sensitivity towards cisplatin in 75% of experiments, and towards 5-FU in 46% of tested cell lines. Western blots suggested that these miRNAs regulate a variety of targets in different resistance-relevant pathways including p53-dependant pathway of apoptosis, DNA-methylation or histone modification. Furthermore, we could prove for the first time Bcl-2 as a direct target of miR-130a-3p and miR-148a-3p, and p53 as a direct target of miR-125a-5p in ESCC. As Bcl-2 and p53 both are part of the p53-dependant pathway of apoptosis, our data suggested that miRNAs target not only several different resistance-relevant pathways, but also different key proteins within a single pathway. This is supported by our findings on the expression of downstream targets Bax and caspases within apoptotic signaling.

Three of the four miRNAs investigated in this study have previously been linked to chemotherapy resistance in a variety of other cancers, with partly contradictory results: miR-125 was shown to increase sensitivity towards Paclitaxel and Cisplatin in cervical cancer [24], or to increase resistance towards Gemcitabine in pancreatic cancer [25]. Similarly, high expression of miR-130a was associated with increasing sensitivity towards Gemcitabine in hepatoma [26], while others described that miR-130a increases resistance towards platinum-based chemotherapy in ovarian cancer [27,28,29,30]. With regards to miR-148a, high miR-148a levels were shown to increase sensitivity towards Docetaxel and Paclitaxel in prostate cancer [31,32], while others did not find any impact of miR-148a expression on resistance towards Gemcitabine in pancreatic cancer [33]. 

The different effects of miRNAs on either cisplatin or 5-FU treatment in our study might be caused by the specific mechanisms of action of these two drugs; 5-FU is a classical anti-metabolite drug. These drugs either inhibit essential biosynthetic processes, or are incorporated into DNA and RNA and impair their normal function. Cisplatin exerts its effects via complex interactions with both nuclear and cytoplasmic signaling pathways. The fact that three of the four selected miRNAs in this study (miR-125a-5p, miR-130a-3p, miR-148a-3p) were demonstrated to directly target Bcl-2 and p53 would be consistent with their main effect being relevant for response to cisplatin rather than 5-FU in the current study.

With regards to tumor biology, we could further demonstrate that all four miRNAs significantly decreased migration, and all but miR-130a-3p decreased adhesion of tumor cells, indicating a significant impact of these miRNAs on the cell’s metastatic potential. In accordance with these findings, all four miRNAs increased apoptosis, and all but miR-148a-3p led to cell cycle arrest in the G1 phase. This fits well with the observation that patients with worse response to neoadjuvant treatment often present signs of more aggressive tumor biology [34,35].

In this context, published data on a potential impact of miR-125a, miR-130a, miR-148a and miR-1226 on migration, adhesion or apoptosis are limited, but existing data support our results and showed that these miRNAs play a role in regulation of metastatic behavior. For example, miR-125a influences migration and invasion of ovarian cancer cells [34], and miR-130a was found to inhibit invasion and migration in breast cancer cells [36]. MiR-1226 seems to regulate apoptosis in human breast cancer cell lines [37], and miR-148a in pancreatic cancer cells [38].

Finally, and most interestingly, we could show that simultaneous manipulation of the expression levels of two miRNAs exhibited an additive sensitizing effect towards cisplatin treatment in 50% (miR-125a-5p/miR-148a-3p) and 75% (miR-148a-3p/miR-130a-3p) of tested cell lines. The missing effect of co-transfections on 5-FU might again be explained by the above-mentioned hypothesis that the selected miRNAs interfere with cisplatin action rather than 5-FU action. 

To our best knowledge, data from co-transfection experiments with miRNAs are fairly rare [39,40,41,42,43] and we identified only two studies that investigated combined effects of two miRNAs on chemotherapy drugs. Zheng and colleagues showed that simultaneous transfection with miR-125b and miR-141 mimics led to an increase in resistance to Taxane-Anthracycline-induced cytotoxicity in breast cancer cell lines [44], and Wang et al. reported that combined inhibition of two of the following miRNAs (miR-125a, miR-125b, miR-205) led to a decrease of Entinostat-induced apoptosis in breast cancer cells [45]. These data are preliminary, but draw a very interesting picture of the complex epigenetic regulation of chemotherapy resistance.

A limiting factor in our study is the fact that this is an in vitro study on cell cultures. The use of cell lines as a pre-clinical model can be limited because it might not reflect person-to-person tumoral heterogeneity in genetic backgrounds, which limits the potential transfer of results into the clinic. However, we tried to address this issue by using six different ESCC cell lines in order to “mimic” interpersonal variability as seen in clinical samples. The fact that some of the miRNAs affected chemotherapy resistance in the majority of cell lines suggests that the observed phenomenon is not limited to a single cell line.

Taken together, our data present strong evidence that specific miRNA signatures of chemotherapy-resistant ESCC cells are responsible for drug resistance and biological aggressiveness of ESCC. Furthermore, our data clearly support the hypothesis that epigenetic regulation of chemotherapy resistance is a complex process with several miRNAs acting additively (and/or antagonistically) via simultaneous regulation of several resistance-relevant pathways on various key proteins within these pathways. In this context, we could demonstrate, for the first time, that miR-130a-3p and miR-148a-3p both target Bcl-2 directly in ESCC, and that miR-125a-5p targets p53 directly in this tumor type. However, the final functional readout of this complex regulatory machinery of chemo-resistance appears to include a set of miRNAs acting together in functional cellular complexes [46].

## 4. Materials and Methods

### 4.1. Cell Culture

ESCC cell lines KYSE-70, KYSE-140, KYSE-180, KYSE-270, KYSE-410 and KYSE-520 (purchased from Deutsche Sammlung von Mikroorganismen und Zellkulturen (DSMZ), Braunschweig, Germany) were cultured using standard media and techniques as described previously [21,22,47].

For experiments on chemotherapy resistance, six cell lines were used. For co-transfection experiments, four cell lines (KYSE-70, KYSE-140, KYSE-270, KYSE-410) were used. For experiments on adhesion, migration, apoptosis and the cell cycle, two cell lines were used (KYSE-270, KYSE-410) were used. For target analyses, three cell lines (KYSE-70, KYSE-270, KYSE-410) were used.

### 4.2. Selection of miRNAs and Modulation of miRNA Expression

We selected a panel of three miRNAs from a characteristic miRNA signature of resistant ESCC cells reported in our earlier study: hsa-miR-125a-5p (#MSY0000443), hsa-miR-130a-3p (#MIN0000425), and hsa-miR-1226-3p (#MSY0005577) (all miRNAs are available from Qiagen, Hilden, Germany). In 5-FU-resistant ESCC cells, both hsa-miR-125a-5p and has-miR-1226-3p were downregulated by 1.92- and 2.45-fold, respectively. In cisplatin-resistant ESCC cells, hsa-miR-130a-3p was 2.28-fold upregulated [19]. We also assessed hsa-miR-148a-3p (#MSY0000243), as we previously demonstrated that this miRNA affects sensitivity towards cisplatin and 5-FU in sensitive and resistant KYSE-410 cells [21]. Of these, miR-125a, miR-130a and miR-148a were also described in a clinical context in gastric cancer patients [48,49,50,51,52].

MiRNA expression levels were manipulated via liposomal transient transfection using Lipofectamine^TM^2000 (Life Technologies, Carlsbad, CA, USA) according to a modified manufacturer´s protocol as described previously [21,47]. MiRNA expression was manipulated in a direction that implies increased sensitivity towards chemotherapy based on previous results. Respective mimics/inhibitors (MSY0000443, MIN0000425, MSY0005577, MSY0000243) and scrambled controls (AllStars Neg. Control siRNA, #1027281) were purchased from Qiagen, Germany.

We further performed co-transfection experiments to investigate combined effects of two miRNAs on drug resistance. Oligonucleotide concentrations and Lipofectamine^TM^2000 doses were adjusted according to results from preliminary experiments (Supplement 1).

### 4.3. qRT-PCR

Successful transfection was confirmed via qRT-PCR (Beckman Coulter, Brea, CA, USA) as described previously [21,47], using SNORD25, SNORD68 and RNU6B (Qiagen, Germany) for normalization (Supplement 2 and 3).

### 4.4. Chemotherapy Treatment and Viability Assay

Chemotherapy treatment with cisplatin and 5-FU was performed 24 h after transfection. After 72 h of chemotherapy, viability assays were performed using MTT assays (Thiazolyl Blue Tetrazolonium Bromide, Sigma-Aldrich, St. Louis, MO, USA) [21,47]. One experiment was performed with four technical replicates, and ten experiments were repeated independently.

### 4.5. Adhesion and Migration Assays

Impact of miRNA modulation on adhesion and migration was investigated using standard assays as described previously [47]. For adhesion, one experiment was performed with 4 technical replicates, and confirmed with five independent experiments. For migration assays, experiments were performed 6 times.

### 4.6. Apoptosis and Cell Cycle Analysis

Impact of miRNA modulation on apoptosis and the cell cycle was investigated 48 h after transfection using standard assays (apoptosis: FITC Annexin V Apoptosis Detection Kit With 7-AAD (BioLegend, London, UK); cell cycle analysis: propidium iodide staining) as per the manufacturer’s protocol and flow cytometry using Beckman Coulter FC500 (Beckman Coulter, Brea, CA, USA). For apoptosis, cells were analyzed via flow cytometry (number of events = 20,000, software for data acquisition: CXP-software by Beckman Coulter) and were distinguished as follows: early apoptotic (Annexin V positive and 7AAD negative) and late apoptotic cells (Annexin V and 7AAD positive); four independent experiments were performed

### 4.7. Target Analysis: Selection of Targets, Western Blotting and Luciferase Assay

We selected potential target genes for the different miRNAs from TargetScan with a Total Context Score of ≤−0.5: Bcl-2-like protein 11, B-cell lymphoma 2, X-linked inhibitor of apoptosis protein (XIAP), mitogen- and stress-activated protein kinase-1 (MSK-1), multidrug resistance protein 1 (MDR1) DNA-(cytosine-)-methyltransferase 1 (DNMT1), peroxisome proliferator-activated receptor gamma (PPARy), human epidermal growth factor receptor 2 (ErbB2/Her2), p53, runt-related transcription factor 3 (RUNX3) and histone deacetylase 4 (HDAC4). We further analyzed downstream targets in the apoptosis pathway (caspase 3 (Casp3), caspase 9 (Casp9) and Bax. Tubulin-β-3 was used as control.

The respective primary antibodies were purchased from BD Bioscience, Heidelberg, Germany (559685, 551097, 610716), Biomol, Hamburg, Germany (A302-747A-T, A303-625A-T), Cell signaling, Danvers, MA, USA (5119, 2435, 2165, 9282, 9662, 9502), BioLegend, UK (653601, 607701, 657407) and Santa Cruz Biochtechnology, Dallas, TX, USA (sc-493). As secondary antibody, we used anti-rabbit-HRP and anti-mouse-HRP (A6154, A9044; Sigma-Aldrich).

Cells were harvested 48 h post-transfection; 15–30 µg of protein were loaded onto 6–10% SDS denaturating polyacrylamide gels. Proteins were transferred onto a PVDF membrane. Membranes were blocked, and primary antibodies were incubated with the membrane overnight at 4 °C. Membranes were washed and incubated with the respective secondary antibody. Blots were developed with the ECL plus Western blotting detection system (Immobilon^TM^, Millipore, Billerica, MA, USA).

Validation of direct targets was performed using luciferase assays. We focused on targets that showed dysregulation at the protein levels after transfection in Western blot analyses. Plasmid isolation from transformed bacteria was done using the Zyppy^TM^ Plasmid Miniprep Kit (Zymo Research, Irvine, CA, USA) according to the manufacturer’s recommendations and the Dual-Glo Luciferase Assay System (Promega, Madison, WI, USA) was used as per the manufacturer’s instructions. Briefly, cells were co-transfected with 20 nmol miRNA mimics and 100 ng of the reporter vector via Lipofectamine^TM^2000, and luciferase activity was measured 24 h after transfection with the Dual-Glo Luciferase Assay System. Activity of firefly and Renilla luciferases were measured as internal controls. Four independent experiments were performed.

### 4.8. Statistical Analysis

All data were presented as means ± standard deviation. The relative cell survival after transfection and chemotherapy treatment was calculated by normalizing the mean corrected absorbance of treated cells to the corresponding controls (given in %). For assessment of the effect of miRNA transfection on sensitivity to chemotherapy, the relative survival of the transfected cells was related to the survival of negative controls, whose cell survival was defined as “zero %”. Data were assessed for statistical significance using parametric and non-parametric tests (Student´s *t*-test and Mann–Whitney *U* test) as appropriate. *p* < 0.05 was considered to be statistically significant. All analyses were performed using SPSS 21.0 (SPSS, Chicago, IL, USA).

## Supplementary Materials

The following are available online at http://www.mdpi.com/1422-0067/19/2/499/s1.

## Abbreviations

BAK1	Bcl-2 homologous antagonist/killer
Bcl-2	B-cell lymphoma 2
BIM	Bcl-2-like protein 11
DNMT1	DNA-(cytosine-)-methyltransferase 1
DSMZ	Deutsche Sammlung von Mikroorganismen und Zellkulturen
ErbB2/Her2	Human epidermal growth factor receptor 2
ESCC	Esophageal squamous cell carcinoma
HDAC4	Histone deacetylase 4
MDR1	Multidrug resistance protein 1
MiRs	microRNAs
MiRNA	microRNA
MSK-1	Mitogen- and stress-activated protein kinase-1
PPARγ	Peroxisome proliferator-activated receptor gamma
R-CHOP	Chemotherapy containing Rituximab, Cyclophosphamide, Hydroxydaunorubicin (also called doxorubicin or Adriamycin), Oncovin (vincristine), Prednisone or Prednisolone
RUNX3	Runt-related transcription factor 3
XIAP	X-linked inhibitor of apoptosis protein
TRAIL	TNF-related apoptosis-inducing ligand
TS	Thymidylate synthase
