# Correction: Complex Epigenetic Regulation of Chemotherapy Resistance and Biology in Esophageal Squamous Cell Carcinoma via MicroRNAs. *Int. J. Mol. Sci.* 2018, *19*, 499

**DOI:** 10.3390/ijms20040921

**Published:** 2019-02-20

**Authors:** Kirsten Lindner, Ann-Kathrin Eichelmann, Christiane Matuszcak, Damian James Hussey, Jörg Haier, Richard Hummel

**Affiliations:** 1 Department of Surgery, University Hospital of Schleswig-Holstein—Campus Lübeck, Ratzeburger Allee 160, 23538 Lübeck, Germany; Kirsten.lindner@uksh.de; 2 Department of General and Visceral Surgery, University Hospital of Münster, Waldeyerstrasse 1, 48149 Münster, Germany; 3 University Cancer Centre Hamburg (UCCH), University Hospital of Hamburg-Eppendorf, Martinistr. 52 (O24), 20246 Hamburg, Germany; C.Matuszcak@uke.de; 4 Department of Surgery, Flinders Medical Centre, Flinders University Adelaide, Flinders Drive, Bedford Park, Adelaide, SA 5042, Australia; damian.hussey@flinders.edu.au; 5 The Nordakademie, Van-der-Smissen Str. 9, 22767 Hamburg, Germany; joerg.haier@nordakademie.de

We would like to submit a correction to the published paper [1]. The reason for the correction lies in the fact that we performed and analyzed a number of experimental studies at the same time, and the current paper was the first publication from this work. However, when analyzing the other studies in detail, we found some inconsistencies; therefore we present the entire analysis of the current manuscript based on the raw data.

In summary, we finally identified a number of minor errors, mostly with regard to the presentation of our data in the original manuscript:

(1) Statistical re-analysis of the experiments shown in Figure 2 revealed an incorrect *p*-value for the miR-148a-3p/miR-130a-3p co-transfection. After correction, the *p*-value changed from *p* ≤ 0.005 to *p* ≤ 0.016 (mentioned in the Abstract, and on page 4). This change does not affect the significance or discussion of results.

In the Abstract we change “Simultaneous manipulation of two microRNAs exhibited additive sensitizing effects towards cisplatin in 50% (miR-125a-5p/miR-148a-3p), and 75% (miR-148a-3p/miR-130a-3p) of cell lines (*p* ≤ 0.006).” to “Simultaneous manipulation of two microRNAs exhibited additive sensitizing effects towards cisplatin in 50% (miR-125a-5p/miR-148a-3p), and 75% (miR-148a-3p/miR-130a-3p) of cell lines (*p* ≤ 0.016).”

In page 4 we change “Co-transfection of miR-148a-3p/miR-130a-3p resulted in significantly increased sensitivity towards cisplatin in all cell lines (+15% to +39%; *p* ≤ 0.005) compared to scrambled controls, and led in 75% of our experiments to an additive effect of co-transfection when compared to transfections with either miRNA alone (Figure 2B).” to “Co-transfection of miR-148a-3p/miR-130a-3p resulted in significantly increased sensitivity towards cisplatin in all cell lines (+15% to +39%; *p* ≤ 0.016) compared to scrambled controls, and led in 75% of our experiments to an additive effect of co-transfection when compared to transfections with either miRNA alone (Figure 2B).”

Figure 2 should be replaced with:

**Figure 2:**
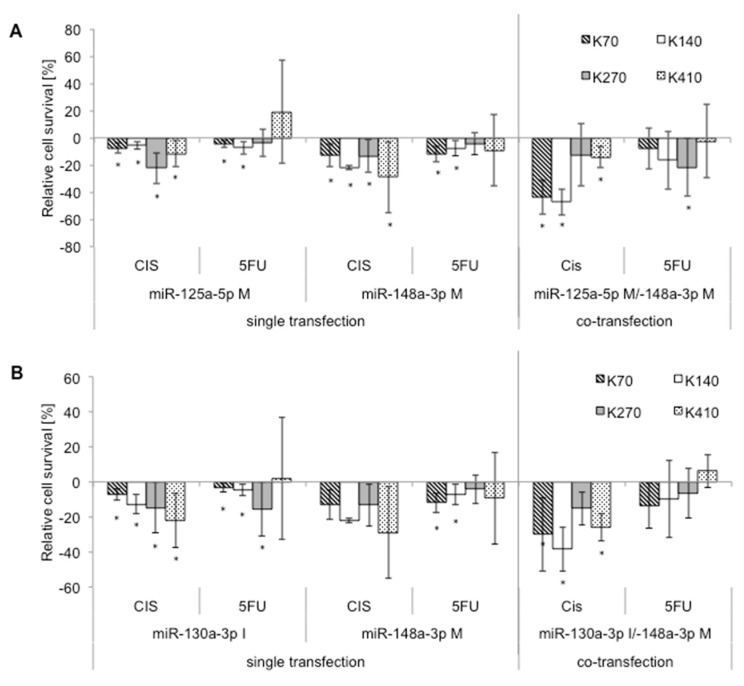
Co-transfection: additive effects of resistance-relevant miRNAs. Effect of co-transfection of miRNAs on cytotoxicity after chemotherapy treatment for: (**A**) miR-125a-5p mimic/miR-148a-3p mimic, and (**B**) miR-130a-3p inhibitor/miR-148a-3p mimic, compared to single transfection controls with each miRNA. Relative cell survival compared to controls given in %, controls set to “zero”. K70: KYSE-70; K140: KYSE-140; K270: KYSE-270; K410: KYSE-410; CIS: cisplatin; 5-FU: 5-fluorouracil; M: mimic; I: inhibitor; and *: significance (*p* ≤ 0.05).

(2) Apoptosis data were found to be partly incorrect due to confusion with data from a second study. Re-analysis, however, confirmed in general most of the results their significance values. In our new analysis we found that early apoptosis after miR-130a inhibition significantly increased, and early apoptosis after miR-148a mimic transfection still increased but failed to reach significance. We adjusted Figure 4 and the corresponding text accordingly. In summary, these changes did not affect the overall significance or discussion of results.

In page 4 we change “Altered expression of all four miRNAs significantly increased (especially late-) apoptosis rates with a maximum increase in apoptosis of up to 332% after miR-125a-5p upregulation (Figure 4).” to “Altered expression of all four miRNAs significantly increased (especially late-) apoptosis rates with a maximum increase in apoptosis of up to 463% after miR-125a-5p upregulation (Figure 4).”

Figure 4 should be replaced with:

**Figure 4:**
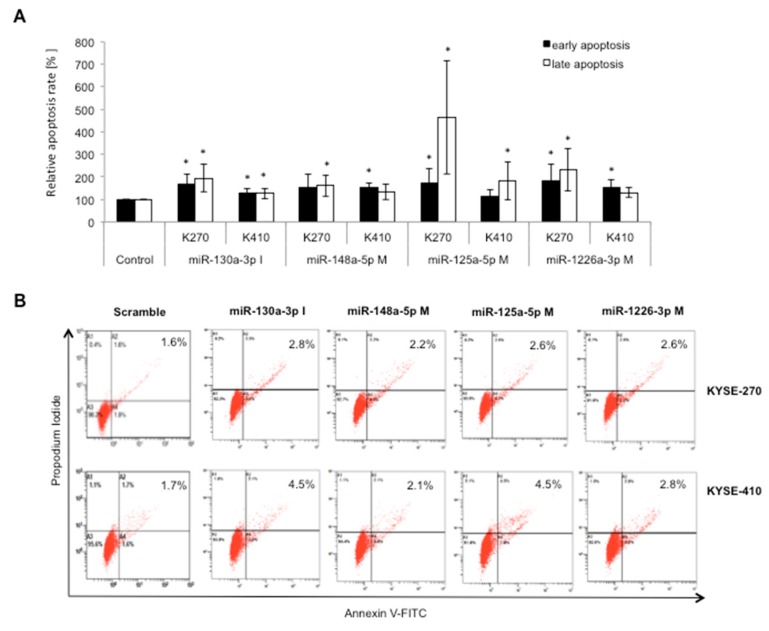
Specific miRNA signatures of resistant cell lines impact on apoptosis in ESCC. (**A**) Relative apoptosis rate of transfected cells vs. negative controls; and (**B**) representative dot plot of the Annexin V-FITC and PI assay on ESCC cells treated with 20 ppmol of different miRNAs after 48 h of transfection. (A1: necrotic cells/false positive cells (Annexin-/7AAD+); A2: late apoptotic cells (Annexin+/7AAD+); A3: viable cells (Annexin−/7AAD−); and A4: early apoptotic cells (Annexin+/7AAD−). K270: KYSE-270; K410: KYSE-410; M: mimic; I: inhibitor; A2: late apoptotic rate; A4: early apoptotic rate; and *: significance (*p* ≤ 0.016).

(3) Protein expression data on XIAP expression after miR-130a downregulation had to be corrected. We found that miR-130a downregulation in fact led, in all experiments, to the upregulation of its putative target. We adjusted Figure 6 and Figure 7 and the corresponding section in the manuscript accordingly.

In page 5 we change “Similarly, PPARγ, Bcl-2, XIAP, and RUNX3 showed decreased protein levels after upregulation of miR-130a-3p.” to “Similarly, PPARγ, Bcl-2, XIAP, and RUNX3 showed increased protein levels after downregulation of miR-130a-3p”.

Figure 6 and Figure 7 should be replaced with:

**Figure 6:**
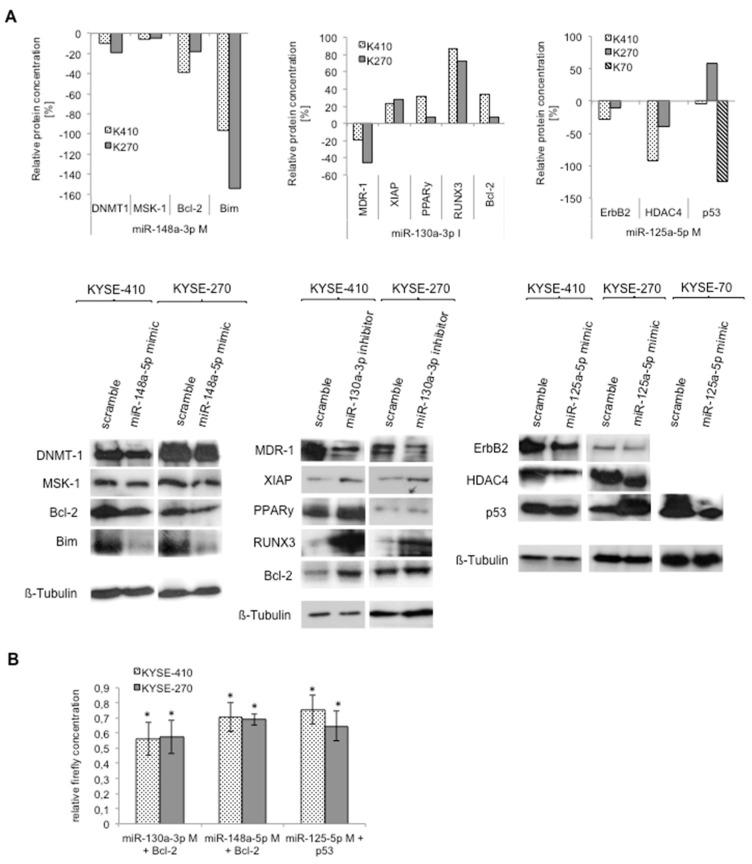
Specific miRNA signatures of resistant cell lines target various resistance-relevant pathways: western blotting and luciferase assays. (**A**) Protein expression of potential targets of respective miRNAs, measured with western blot in KYSE-410 and KYSE-270 cells (and in case of p53 analysis in KYSE-70 cells); and (**B**) luciferase assay after miRNAs transfection. Relative firefly concentration of target protein of interest was measured with the Dual Glo Luciferase Kit 24 h after transfection with miRNA precursor molecules. DNMT-1: DNA (cytosine-5)-methyltransferase 1; MSK-1: mitogen and stress activated protein kinase 1; Bcl-2: B-cell lymphoma 2; MDR1: multidrug resistance protein 1; XIAP: X-linked inhibitor of apoptosis protein; RUNX3: Runt-related transcription factor 3; PPARy: Peroxisome proliferator-activated receptor gamma; HDAC4: Histone deacetylase 4; ErbB2: Receptor tyrosine-protein kinase; K270: KYSE-270, K410: KYSE-410, K70: KYSE-70; M: mimic; I: inhibitor; Scr: Scramble; and *: significance (*p* ≤ 0.05).

**Figure 7:**
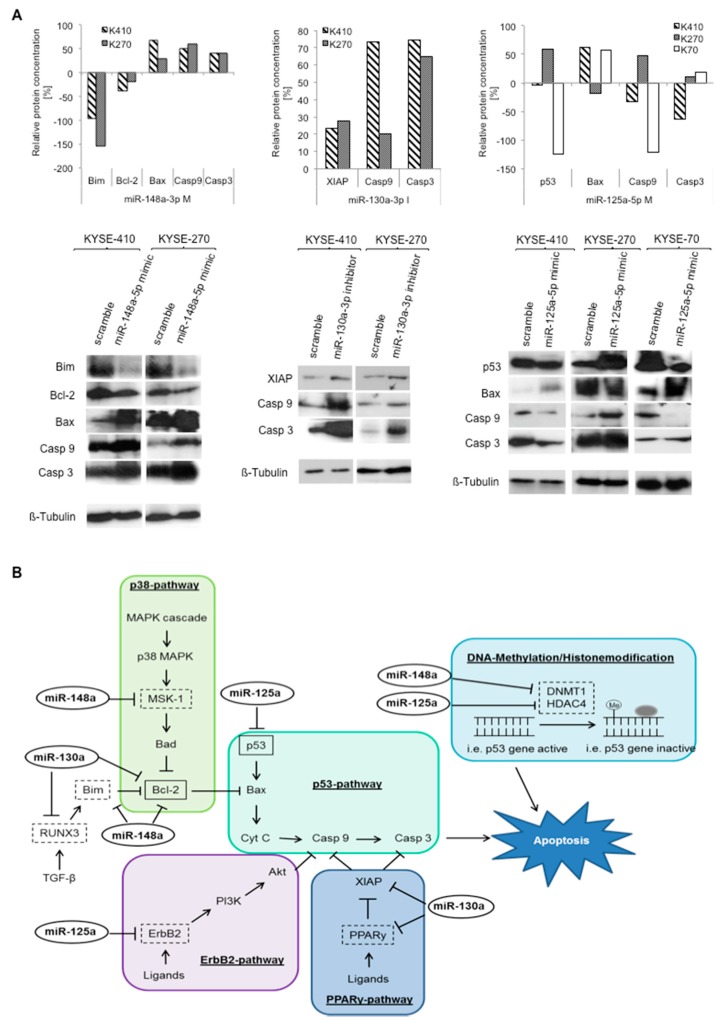
Specific miRNA signatures of resistant cell lines target various resistance-relevant pathways: pathway analyses. (**A**) Effect of miRNA transfection on the p53-dependant apoptosis pathway in ESCC cells. Relative protein expression levels of pathway compounds compared to controls were measured via western blot analysis; and (**B**) overview of the complex process of miRNA-mediated regulation of several resistance-relevant pathways at various key spots. Confirmed direct targets are marked in boxes with solid lines, potential targets are marked in boxes with dotted lines (adapted from Krammer et al. [20]); Bcl-2: B-cell lymphoma 2; Bax: Bcl-2-associated X protein; Casp: caspase; XIAP: X-linked inhibitor of apoptosis protein; K270: KYSE-270; K410: KYSE-410; K70: KYSE-70; M: mimic; I: inhibitor; Scr: Scramble; and *: significance (*p* ≤ 0.05).

In summary, these changes did not impact in any way the significance of the overall results or the conclusions of our paper. We updated the manuscript, and the original version will remain online. We apologize for any inconvenience we may have caused to our readers.
