# Supplementary material for: Complex Epigenetic Regulation of Chemotherapy Resistance and Biology in Esophageal Squamous Cell Carcinoma via MicroRNAs
Source: Int J Mol Sci. 2018 Feb 7;19(2):499. doi: 10.3390/ijms19020499 (PMC5855721; doi:10.3390/ijms19020499)
Supplement: Supplementary file 1 [file ijms-19-00499-s001.pdf]

**Supplementary Materials:**

Supplement 1: Details to "Methods"

Supplement 2: Validation of miRNA expression after transfection via qRT-PCR.

Supplement 3: Adjustment of Oligonucleotide concentrations and Lipofectamine™2000. Influence of used dosage of miRNA mimics on miRNA expression measured by qRT-PCR after single vs. double transient transfection (10 nm oligonucleotide and 20 nm oligonucleotides) for miR-125a-5p/miR-148a-3p (A) and miR-130a-3p/miR-148a-3p (B). M: Mimic, I: Inhibitor; MV: mean value; SD: Standard Deviation; K410: KYSE-410, K270: KYSE-270; miR: miRNA.

**Supplement 1**

Modulation of miRNA expression

Cells were plated in 6-well plates in medium at a density of $2x10^4$ cells/well and allowed to attach for 24 h. At a confluence of 40-50%, medium was changed to Opti-MEM® Reduced Serum Media (Invitrogen, Carlsbad, CA) and cells were transfected for 24 h with 20 nanomol oligonucleotides using Opti-MEM® to prepare oligonucleotide-Lipofectamine™2000 complexes.

RNA extraction and qRT-PCR

For qRT-PCR cells were lysed after transfection using TRIzol® reagent (Invitrogen Life Technologies, NY, USA) according to the instructions of the manufacturer. Total miRNA was extracted using the miRNeasy Kit (Qiagen, Germany) according to manufacturer´s protocol. MiRNA quality and concentration were determined using UV spectrophotometry. miScript system (Qiagen, Germany) was used for qRT-PCR. Briefly, for each sample, 500 ng of total RNA was used for reverse transcription into cDNA using miScript II Reverse Transcription Kit (Qiagen, Germany) according to manufacturer´s protocol. Real-time PCR were performed using miScript SYBR® Green PCR Kit (Qiagen, Germany) according to manufacturer's protocol. All samples were assayed in triplicate reactions using BioRad CFX 384 Real-Time System (Hercules/California USA). Quantitative analysis was performed using Bio-Rad CFX Manager 2.1.

Chemotherapy treatment and viability assay

Transfected cells were seeded 24 hours post-transfection onto 96-well plates and allowed to attach for 24 hours to reach confluence of about 50%. Then, cisplatin or 5-FU was freshly prepared and added. The concentrations of drugs represented the approximate IC50 doses after 72-hour exposure as determined in previous experiments (data not shown). After 72 hours of incubation with chemotherapy, viability assays were performed using MTT assays (Thiazolyl Blue Tetrazolonium

Bromide, Sigma-Aldrich, St. Louis, USA). 100 µL MTT solution (1 mg/mL MTT in cell culture medium) was added per well. After two hours, the supernatant was removed and MTT formazan crystals were solubilized in 100 µL 2-Propanol (AppliChem, St. Lois, USA) per well. Absorbance at 570 nm was measured on the spectrophotometer Dynatech MR5000 (Dynatech, Ashford, UK) using the software MikroWin2000 (Mikrotek Laborsysteme, Overath, Germany).

Adhesion and Migration assays

Cells were trypsinized and underwent a 60 minutes reconstitution period using serum free medium containing 1% albumin. For adhesion assays, cells were transferred to 96-well plates (30.000 cells/well) coated with collagen type I and fibronectin (Corning B.V. Life Sciences, Amsterdam, Netherlands). Cells were plated under the stimulation of 10% fetal bovine serum, and adhesion was assessed after 30, 60, and 90 minutes using crystal violet staining and quantified by photospectrometer. One experiment was performed with 4 technical replicates, and confirmed in five independent experiments. For migration assays, cells were plated onto the upper chamber of a 24-well Boyden chamber coated with collagen type I and fibronectin with a 8 µm pore polycarbonate membrane in medium without serum, and medium containing 10% fetal bovine serum was filled in the lower chamber as chemo-attractant. Cells that did not migrate through the pores were removed after 18 hours using cotton swabs. Membranes were stained using crystal violet, and migrated cells were counted in 9 gridded high-power fields per membrane under an inverted microscope. Assays were performed in 6 independent experiments.

## Supplement 2

| miR | K70 | | | K140 | | | K180 | | | K270 | | | K410 | | | K520 | | |
|---|---|---|---|---|---|---|---|---|---|---|---|---|---|---|---|---|---|---|
| | BL | Transfection | | BL | Transfection | | BL | Transfection | | BL | Transfection | | BL | Transfection | | BL | Transfection | |
| | | MW | STD | | MW | STD | | MW | STD | | MW | STD | | MW | STD | | MW | STD |
| miR-125a | 22.42 | 357.34 | 39.52 | 22.78 | 737.75 | 113.20 | 23.7 | 1107.95 | 76.71 | 24.16 | 2002.97 | 88.33 | 21.18 | 1364.51 | 113.56 | 22,74 | 450.87 | 48.84 |
| miR-130a | 23.85 | 0.36 | 0.07 | 23.45 | 0.63 | 0.11 | 23.63 | 0.34 | 0.07 | 24.27 | 0.31 | 0.04 | 23.03 | 0.71 | 0.06 | 23.17 | 0.52 | 0.04 |
| miR-148a | 24.75 | 1256.26 | 109.26 | 23.36 | 1575.00 | 157.00 | 26.61 | 1578.26 | 145.24 | 24.09 | 308.59 | 25.71 | 26.66 | 3567.47 | 280.73 | 25.06 | 2544.86 | 273.40 |
| miR-1226 | 25.7 | 12967.07 | 1064.97 | 25.18 | 13500.66 | 4115.00 | 23.21 | 1726.00 | 324.00 | 24.44 | 7201.92 | 299.45 | 25.5 | 627.69 | 21.82 | 21.13 | 464.77 | 55.92 |

Transfection was performed using miR-125a Mimic, miR-130a Inhibitor, miR-148a Mimic and miR-1226 Mimic, respectively.
miR=microRNA, K=squamous cell carcinoma cell line KYSE, BL=baseline, MW= mean value after transfection, STD=standard deviation

## Supplement 3

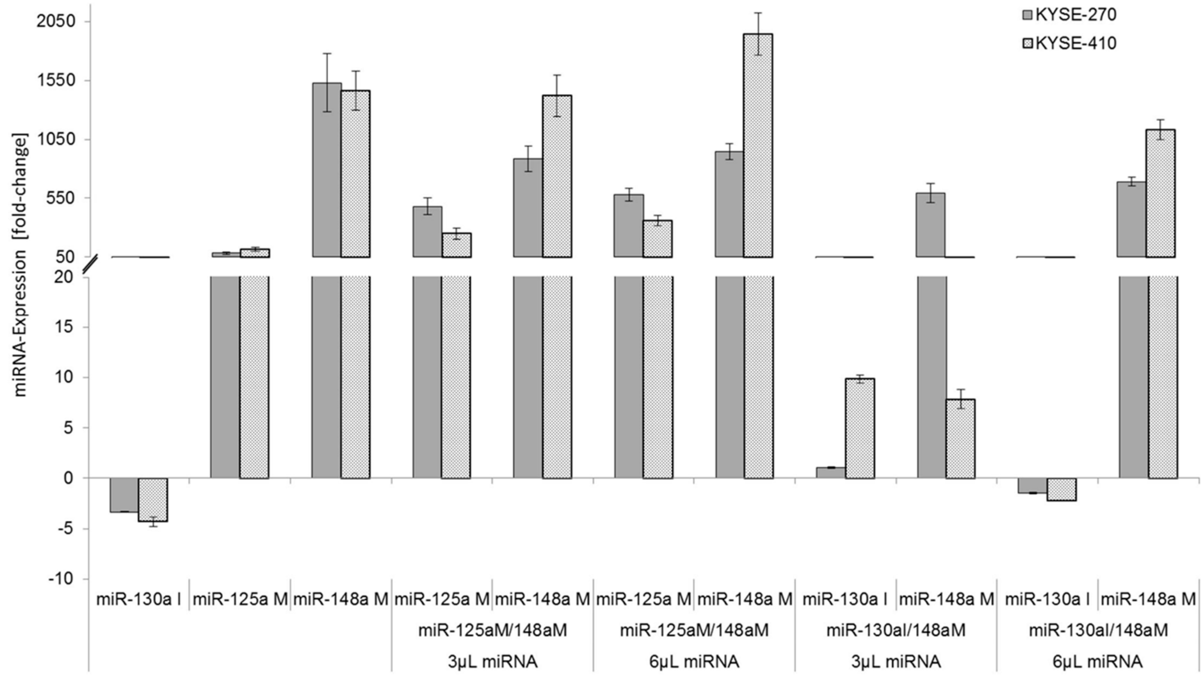


| | | | | KYSE-270 | | KYSE-410 | |
|---|---|---|---|---|---|---|---|
| | µL miRNA per transfec-tion | miRNA pair | Mimic/Inhibitor | Mean value | Standard deviation | Mean value | Standard deviation |
| single transfection | | | miR-130a I | -3,33 | 0,028 | -4,31 | 0,5 |
| | | | miR-125a M | 83,31 | 8,6 | 114,75 | 17,85 |
| | | | miR-148a M | 1530,66 | 246,57 | 1462,44 | 167,82 |
| double transfection | 3 | miR-125aM/148aM | miR-125a M | 480,20 | 70,57 | 248,46 | 50,29 |
| | | | miR-148a M | 884,30 | 110,07 | 1420,00 | 176,46 |
| | 6 | miR-125aM/148aM | miR-125a M | 580,62 | 52,9 | 360,85 | 45,26 |
| | | | miR-148a M | 945,47 | 65,28 | 1942,16 | 180,06 |
| | 3 | miR-130aI/148aM | miR-130a I | 1,09 | 0,08 | 9,87 | 0,39 |
| | | | miR-148a M | 592,12 | 79,46 | 7,85 | 0,96 |
| | 6 | miR-130aI/148aM | miR-130a I | -1,46 | 0,06 | -2,22 | 0,06 |
| | | | miR-148a M | 691,00 | 36,26 | 1132,07 | 85,72 |
